# Enabling Web-scale data integration in biomedicine through Linked Open Data

**DOI:** 10.1038/s41746-019-0162-5

**Published:** 2019-09-10

**Authors:** Maulik R. Kamdar, Javier D. Fernández, Axel Polleres, Tania Tudorache, Mark A. Musen

**Affiliations:** 1 Center for Biomedical Informatics Research, Stanford University, Stanford, CA USA; 2 Vienna University of Economics & Business, Vienna, Austria; 3 Complexity Science Hub Vienna, Vienna, Austria

**Keywords:** Databases, Data integration, Databases, Computational platforms and environments

## Abstract

The biomedical data landscape is fragmented with several isolated, heterogeneous data and knowledge sources, which use varying formats, syntaxes, schemas, and entity notations, existing on the Web. Biomedical researchers face severe logistical and technical challenges to query, integrate, analyze, and visualize data from multiple diverse sources in the context of available biomedical knowledge. Semantic Web technologies and Linked Data principles may aid toward Web-scale semantic processing and data integration in biomedicine. The biomedical research community has been one of the earliest adopters of these technologies and principles to publish data and knowledge on the Web as linked graphs and ontologies, hence creating the Life Sciences Linked Open Data (LSLOD) cloud. In this paper, we provide our perspective on some opportunities proffered by the use of LSLOD to integrate biomedical data and knowledge in three domains: (1) pharmacology, (2) cancer research, and (3) infectious diseases. We will discuss some of the major challenges that hinder the wide-spread use and consumption of LSLOD by the biomedical research community. Finally, we provide a few technical solutions and insights that can address these challenges. Eventually, LSLOD can enable the development of scalable, intelligent infrastructures that support artificial intelligence methods for augmenting human intelligence to achieve better clinical outcomes for patients, to enhance the quality of biomedical research, and to improve our understanding of living systems.

## A data deluge in biomedicine

The 21st century is the age of data and knowledge explosion in biomedicine. Several key events, such as the completion of the Human Genome Project and the advent of next-generation sequencing technologies,^1,2^ the enactment of the Health Information Technology for Economic and Clinical Health (HITECH) Act,^3^ and the Internet of Things phenomenon,^4^ have led to a significant increase in the volume, velocity, and variety of biomedical data. To create a complete profile of any individual, to perform predictive and inferential analytics, and to investigate the mechanisms behind any biological event, the biomedical researcher has at his disposal several different sources of data: medical records, imaging data (e.g., X-ray images, MRI scans), claims, sequencing data (e.g., gene expression, DNA methylation, MicroRNA expression, chromatin accessibility data), genotypes, sensor data (e.g., wearable data, social media streams).

There is also a rapid increase in the number of structured, machine-processable knowledge artifacts, as well as an increase in unstructured knowledge sources in the form of publications in biomedicine. Knowledge bases (e.g., DrugBank,^5^ UniProt^6^) and ontologies (e.g., Gene Ontology,^7^ National Cancer Institute Thesaurus^8^) are widely-used and popular resources in biomedicine, and contain knowledge pertaining to molecules (e.g., drugs, proteins) and their characteristics, biological pathways, animal models and phenotypes, organs, symptoms, diseases, and adverse reactions.^9,10^ As of January 2019, there are more than 750 ontologies and terminologies in BioPortal,^11^ the world’s most comprehensive repository of biomedical ontologies. MEDLINE,^12^ the largest repository of scientific articles in biomedicine and the primary component of the PubMed search engine,^13^ currently contains more than 25 million citations and thousands more are added each day.

Despite the open availability of many important databases and knowledge bases, biomedical researchers still face severe logistical and technical difficulties when integrating, analyzing and visualizing heterogeneous data and knowledge from these diverse and isolated sources. These tasks pose a steep learning curve for most biomedical researchers. Researchers need to be aware of the sources where the data and knowledge relevant to their research exist. Depending on the availability and the accessibility, biomedical researchers need exhaustive computational resources and extensive programming skills to query and explore the data and knowledge sources. The heterogeneity across these sources, in terms of formats, syntaxes, notations and schemas, severely stymies the systematic consumption of data and knowledge stored in these sources. The biomedical researcher ends up learning multiple systems, configurations and access requirements, significantly increasing the complexity and time of scientific research. In most cases, the researcher just hops across web portals and search engines (e.g., PubMed^13^) to retrieve relevant data pertaining to their unique requirements or to retrieve answers to queries, such as “What are the medications prescribed to melanoma patients that have a V600E mutation in their BRAF gene?”.

While we are on the cusp of another artificial intelligence revolution in biomedicine^14^ with the development of advanced machine learning methods that can analyze several modes of data, scalable intelligent infrastructures that can support these methods are not yet prevalent. These infrastructures must provide integrated biomedical data and semantically-interlinked entities for seamless utilization in machine learning methods. With such a confluence, biomedical researchers can then mine novel associations from multiple, diverse, and heterogeneous sources simultaneously in the context of all relevant knowledge to achieve better clinical outcomes for individuals on a personalized basis, to enhance the quality of biomedical research, and to improve our understanding of living systems.

Semantic Web and Linked Open Data are promising solutions that can be used to develop such scalable infrastructures for complex biomedical tasks. Web-scale Semantic Processing and Data Integration is the methodology through which biomedical researchers can query, retrieve, integrate, and analyze data and knowledge from multiple sources on the Web without the requirement on the part of the researchers to download and manually integrate those sources.^15^ Ideally, the researchers should not be concerned with the location, heterogeneous schemas, syntaxes, varying entity notations and representations of the underlying sources, or the mappings to reconcile similar concepts, relations, and entities between these sources. Integrated content can then be used in machine learning platforms to drive biomedical research and discovery, as well as improve clinical outcomes of individuals.

In this paper, we will present an overview of the opportunities proffered by Semantic Web technologies and the Life Sciences Linked Open Data (LSLOD) cloud to enable Web-scale semantic processing and to develop applications that integrate data and knowledge from multiple heterogeneous sources in different biomedical domains. We will provide our perspective on the challenges associated with querying and consuming data and knowledge from multiple LSLOD sources in an integrated fashion, which are faced by most biomedical researchers. Finally, we will provide a few technical solutions that address these challenges and that can assist software engineers and biomedical researchers to develop the next generation of intelligent infrastructures to power advanced machine learning methods.

## Life Sciences Linked Open Data (LSLOD) cloud

The Linked Open Data (LOD) cloud has emerged from the vision of a Web of data that co-exists with the current Web of documents.^16^ The World Wide Web Consortium (W3C) has recommended and standardized a set of Semantic Web languages and technologies that aim toward accomplishing specific tasks for the creation of this Web of data and knowledge. We present a brief technical overview on Uniform Resource Identifiers (URIs), the Resource Description Framework (RDF)^17^ and Linked Data principles^18^ for representing and linking data on the Web as graphs in Box 1, on RDF Schema^19^ (RDFS) and the Web Ontology Language^20^ (OWL) for defining Web-based vocabularies and ontologies in Box 2, and the SPARQL graph query language^21^ to query multiple diverse RDF graphs in Box 3.

**Fig. 1:**
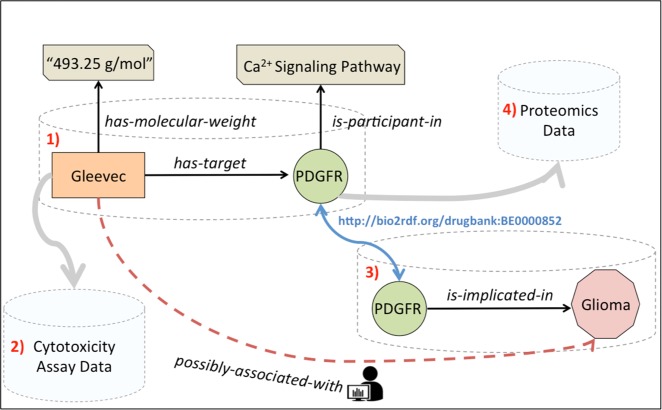
Diagrammatic representation of Linked Data and knowledge. RDF facilitates representation and data merging by extending the linking structure of the web. Entities in different sources (e.g., PDGFR protein in Source 1 and Source 3) are represented using a unique URI. Disparate sources can have independent facts (or triples) such as (Gleevec, has-target, PDGFR) and (PDGFR, is-implicated-in, Glioma), or other data (e.g., molecular weight of drugs, pathway information for proteins) that can be easily linked and integrated using RDF. A human user or a computational agent should, ideally, be able to navigate this Web of data to generate novel hypotheses (e.g., (Gleevec, possibly-associated-with, PDGFR)) and discover relevant data and knowledge in other sources (e.g., cytotoxicity assay data in Source 2)

Using a hypothetical scenario from biomedicine, we will provide an intuitive explanation on what it means for data and knowledge to be linked and queried on the Web (Fig. 1). Suppose a researcher wishes to retrieve and integrate all available data and knowledge related to a given Drug entity (e.g., drug–protein target interactions, downstream targets located in biological pathways, publications that describe the drug, assays that test the cytotoxicity of the “drug active ingredient”). In the current state of art, biomedical data and knowledge exist on the Web in fragmented and isolated sources (e.g., in relational databases, flat files, or graph databases) that may or may not provide programmatic access to users. Consider that two imaginary isolated sources, a drug-related knowledge base (Source 1) and a biological pathway or disease-related knowledge base (Source 3) exist on the Web. The facts Gleevec $\mathop{\longrightarrow}\limits_{{has - target}}$ PDGFR (platelet-derived growth factor receptor) and PDGFR $\mathop{\longrightarrow}\limits_{{is - implicated - in}}$ Glioma may exist in Source 1 and Source 3, respectively. Such facts are not always necessarily represented as directed edges—for example, these facts may be represented using cell values in a database table. Similarly, other arbitrary databases (e.g., Source 2 contains cytotoxicity assay data and Source 4 contains proteomics data) that may be relevant for the researcher also exist on the Web.

**Fig. 2:**
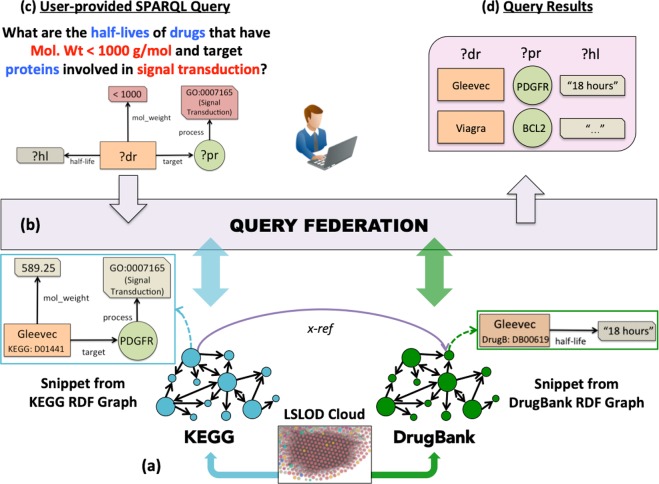
SPARQL Query Federation. **a** Two sources—KEGG, a knowledge base of biochemical pathways, and DrugBank, a database containing molecular characteristics of drugs, are available as RDF Graphs on the LSLOD cloud (The LSLOD cloud image is derived with permission under a CC-BY Attribution 4.0 International Licence from the LOD cloud diagram at lod-cloud.net after cropping modifications). Snippets of the KEGG and DrugBank RDF graphs are respectively shown, and similar Drug entities in these RDF graphs are mapped using the ‘x-ref’ link. **b** An intelligent query federation architecture can determine which SPARQL endpoint to query based on the content of the underlying RDF graphs (i.e., drug–protein interaction knowledge from KEGG, and half-life information from DrugBank). **c** The user-provided query is shown using a visual SPARQL representation, with variable nodes ?dr (drugs), ?pr (proteins), and ?hl (half-lives of drugs). This query is executed by the user against the query federation architecture. **d** The query federation architecture returns a result set to the user (e.g., Gleevec targets PDGFR, and has a half-life of “18 h”)

Using RDF (Box 1), these facts will be represented as triples (i.e., directed edges) in a network of entities represented using URIs. It is assumed that publishers, who convert their data to RDF graphs, will either reuse from a uniform set of URIs (e.g., shared PDGFR entity URI drugbank:BE0000852 between Source 1 and Source 3 in Fig. 1), or map similar entities through their URIs (using entity reconciliation mapping services) and those mappings are present on the LOD cloud as physical links. These links, often called cross-reference or ‘x-ref’ links, between two URIs in different LSLOD sources usually indicate that the represented entities are similar (e.g., drugbank:DB00619 $\mathop{\longleftrightarrow}\limits_{{x - ref}}$ kegg:D01441 in Fig. 2 indicates similar Gleevec drug entities present in different sources). In an ideal sense, the boundaries between different sources will vanish and a Web of Data composed of interlinked entities will manifest. A human user or a computational agent can explore this linked Web of Data by just navigating the different URIs (similar to how a user navigates on the World Wide Web using the URLs of web pages), and generate novel hypotheses (e.g., a naïve link prediction method may indicate Gleevec $\mathop{\longrightarrow}\limits_{{possibly - associated - with}}$ Glioma, since Glioma can be navigated from Gleevec via the PDGFR entity URI and the semantics of the edges connecting the different entities in Fig. 1).

While the biomedical researcher can navigate across the Web of interlinked biomedical entities and data, the SPARQL graph query language and a query federation architecture can be used for formulation of queries that target a set of RDF graphs on this Web (Box 3). The process of SPARQL query federation is depicted in Fig. 2. Consider that the biomedical researcher wishes to retrieve the list of Drug entities (and their half-lives) that have molecular weight <1000 g/mol and target Protein entities involved in the Signal Transduction process. A visual representation of the SPARQL query is depicted in Fig. 2c. For this query, multiple RDF graphs need to be queried as no single source may contain the relevant information. Two sources—DrugBank^5^ and the Kyoto Encyclopedia of Genes and Genomes (KEGG)^22^ are present as RDF graphs on the LOD cloud (Fig. 2a). Similar Drug entities with different URIs in these sources are mapped to each other using ‘x-ref’ links (e.g., Gleevec). An “intelligent” SPARQL query federation architecture determines to query the DrugBank RDF graph for half-life information, and the KEGG RDF graph for knowledge on drug–protein interactions and biological pathways (Fig. 2b). The query retrieves tuples (Gleevec, PDGFR, “18 h”) and (Viagra, BCL2, “4 h”) as query results for three variables: (i) Drug ID/Label, (ii) Protein ID/Label, and (iii) Half-life of the drug (Fig. 2d). A few benefits of Web-based SPARQL query federation approach over conventional approaches for data integration (e.g., data warehousing) are listed in Box 3.

Since 2006, using Semantic Web technologies and Linked Data principles, more than 1200 distinct data and knowledge sources from different research areas in life sciences, economics, geography, linguistics, government, media, etc. have been published and linked on the LOD cloud. A representative LOD cloud diagram is shown at https://www.lod-cloud.net/.

The challenges stemming from the integration of disparate, heterogeneous biomedical data and knowledge sources on the Web have led biomedical publishers to be some of the earliest adopters of Semantic Web technologies and Linked Data principles.^10,23–35^ The various biomedical data and knowledge sources published and linked using Semantic Web technologies are often collectively referred to as the Life Sciences Linked Open Data cloud (LSLOD).^25,36^ A few of these biomedical initiatives that use Semantic Web technologies are listed in Table 1. While historically, Semantic Web developers have transformed existing open sources to RDF graphs and OWL ontologies, data providers themselves are now embracing Semantic Web technologies and provide content formalized using RDF or OWL (e.g., NIH PubChem RDF^37^), or even incorporate SPARQL functionality in their Web portals (e.g., the European Bioinformatics Institute RDF Platform^28^). From the perspective of a biomedical researcher, Semantic Web technologies and the LSLOD cloud may have potential advantages for Web-scale computation, seamless integration of big biomedical data and knowledge, and structured querying and reasoning over multiple heterogeneous sources simultaneously (i.e., Web-scale semantic processing and integration).

**Table 1:** Examples of Popular LSLOD sources

LSLOD Source	Description
Bio2RDF	Network of Linked Data resources generated from heterogeneously formatted sources published by multiple data providers (e.g., DrugBank—molecular characteristics of drugs, KEGG—drug–protein interactions and biological pathways, PharmGKB—pharmacogenomics knowledge)
BioPortal	An open online repository of biomedical ontologies, with more than 750 biomedical ontologies and terminologies (as of January, 2019), available for querying via SPARQL. Popular ontologies include Gene Ontology—used for enrichment analysis during microarray experiments, SNOMED CT – used for electronic exchange of clinical information, and ChEBI ontology used for annotation of molecular entities.
European Bioinformatics Institute (EBI) RDF Platform	SPARQL access to their proprietary databases (e.g., UniProt—protein sequences and annotations, ChEMBL—bioactive molecules, and Reactome—biological pathways)
PubChem RDF	PubChem data repository containing data on substances, compounds, structures, and biological assays, published as Linked Data
WikiPathways	Database of biological pathways maintained using a crowdsourcing architecture
NLM MeSH	Medical Subject Headings (terms used to index publications) represented as RDF
Linked TCGA	DNA methylation, gene expression and clinical data of cancer patients from The Cancer Genome Atlas
PathwayCommons	Data warehouse (HPRD, MiRTarbase, BioGrid, IntAct, etc.) of pathway and molecular interaction databases
DisGenet	Data warehouse (ClinVar, EXAC, dbSNP, GWAS Catalog, etc.) on genes and variants associated to human diseases
NextProt	Data warehouse (IntAct, Peptide Atlas, COSMIC, etc.) on human proteins, structures and interactions
Wikidata	A collaboratively edited knowledge base consisting of structured Wikipedia data, including data relevant for biomedicine

Box 1 Resource Description Framework (RDF) RDF is a simple, standard triple-based model for data interchange and representation on the Web.^17^ Each entity (e.g., Gleevec) or a class of entities (e.g., Drug) is considered to be a “thing” or a “resource”, that is represented using a Uniform Resource Identifier (URI). An example of an HTTP URI is http://bio2rdf.org/drugbank:DB00619, where http://bio2rdf.org/drugbank: is the URI namespace and DB00619 is the identifier of the drug Gleevec.

Using RDF, the relations will be expressed as [subject, predicate, object] triples. Each component of this triple (i.e., subject, predicate, or object) is represented using an URI. Hence, RDF extends the linking structure of the Web by using the URIs to represent relations between two resources. This facilitates integration and discovery of relevant data and knowledge even if the schemas and syntaxes of the underlying data sources differ. RDF allows structured and semi-structured data to be mixed, exposed, and shared across different applications. If the isolated sources in Fig. 1a are transformed to RDF, the different entities in these relations will be represented as unique HTTP URIs. Hence, [Gleevec, has-target, PDGFR] will be a valid triple in the RDF Source 1, where each component is represented using an URI (e.g., PDGFR entity URI drugbank:BE0000852).

To ‘dereference’ a URI means to convert a relative URI reference to an absolute form by attempting to obtain a representation of the resource that it identifies. Dereferencing any URI enables the user to discover additional data and knowledge on the LOD cloud related to the representative entity. For example, dereferencing the Gleevec URI (http://bio2rdf.org/drugbank:DB00619) using any Web browser (e.g., Google Chrome) will provide additional information on the entity Gleevec retrieved from other relevant sources (e.g., cytotoxicity assay data, molecular weight, protein targets of Gleevec).

Linked Data principles: To ensure the quality of the data and knowledge sources published using RDF, the W3C has established the following four principles for publishing Linked Data:^18^ [noitemsep]

1. Use URIs as names for entities.
2. Use HTTP URIs so that people can look up those entities using a Web browser.
3. Provide useful information when someone looks up a URI (i.e., dereferenceable HTTP URIs).
4. Include RDF statements that link an entity to other URIs so that users can discover related information regarding that entity (reuse and linking).

Box 2 RDF Schema (RDFS) and Web Ontology Language (OWL) RDF is essentially only a triple-based, schema-less modeling language. The schema of an RDF dataset is represented using secondary specifications such as RDFS^19^ or OWL.^20^ RDFS and OWL enable publishers to define structured Web-based vocabularies and ontologies that enable richer integration and interoperability of data among descriptive communities. Such an independent representation facilitates the evolution and modularization of the schemas separately from the data.

RDFS facilitates the modeling and inclusion of instantiation triples (e.g., [Gleevec, type, Anti-neoplastic Drug]), and classification triples of the types subClassOf (e.g., [Anti-neoplastic Drug, subClassOf, Drug]) and subPropertyOf (e.g., [inhibit, subPropertyOf, has-target]). All entities within a class share similar characteristics, such as attributes and relations. RDFS also provides annotation properties that can aid publishers to include human-readable annotations for different entity and property URIs (e.g., drugbank:DB00619 URI has a label ‘Gleevec’ and a description “Imatinib is a small molecule kinase inhibitor used to treat certain types of cancer. It is currently marketed by Novartis as Gleevec (USA) or as its mesylate salt, imatinib mesilate (INN).”)

OWL extends the capabilities of RDFS and facilitates the inclusion of advanced class expressions, often composed of logical operators (e.g., A class expression [Agonist Drug ∪ Antagonist Drug] with the union operator ∪ indicates a new class composed of Agonist Drug entities and Antagonist Drug entities) and property restrictions (e.g., [Drug, subClassOf, Compound ∩ has-target some Protein] indicates that a Drug entity is a Compound entity that targets at least one Protein entity). OWL documents, known as ontologies, can also be published on the LOD cloud and may refer to or to be referred (i.e., reused) from other OWL ontologies and Linked Data resources. Knowledge expressed in RDFS vocabularies and OWL ontologies can be exploited by computer programs, called reasoners, to verify the consistency of that knowledge (e.g., a Protein entity implicated in two biological processes that can not happen at the same time) or to make implicit knowledge explicit and to generate novel inferences (e.g., all members of a particular drug class target at least one protein involved in Signal Transduction through subClassOf and role restriction expressions).

It should be noted that all class and property mentions in these above examples are essentially URIs. Data and knowledge publishers are expected to adhere to the standard best practices (e.g., Linked Data principles and ontology engineering best practices^83^) when using these URIs to represent classes and properties in their RDFS vocabularies and OWL ontologies (Gleevec may exist as an instance of the class Drug in one source, or as a separate class such that [Gleevec, subClassOf, Drug] may exist as a triple in another source on the LOD cloud. This is an example of semantic mismatch).

Box 3 SPARQL Protocol and RDF Querying Language (SPARQL) The Linked Open Data (LOD) cloud consists of different data and knowledge sources, published as directed graphs using the RDF triple-based model and linked with each other (ideally) through reuse of different URIs, with schemas described using the RDFS and OWL languages. The SPARQL graph query language can facilitate users to query multiple diverse RDF graphs, as well as the RDFS vocabularies and OWL ontologies, exposed through SPARQL endpoints in the LOD cloud.^21^

Each SPARQL query is composed of triple patterns. A triple pattern is essentially similar to an RDF triple, but has a variable node (i.e., ?x) in at least one of the subject, predicate or the object components of the triple. For example, [?x, has-target, PDGFR] triple pattern will retrieve all drugs that target the protein entity PDGFR. SPARQL also supports (i) extensible value testing (e.g., retrieve Drug entities with exactly one target), (ii) filtering of literals (e.g., retrieve Drug entities with molecular weight less than 500 g/mol), and (iii) constraining queries by source RDF graph (e.g., retrieve Drug entities where the drug–protein target relation is present only in DrugBank source). In some cases, SPARQL can be combined with an ontology reasoner for semantic query expansion–for example, the query ‘Retrieve Drug entities that target Protein entities involved in Signal Transduction’ will retrieve drug entities related to Apoptotic Signaling Pathway and Necroptotic Signaling Pathway, since both these classes will be children classes of Signal Transduction. Multiple triple patterns can be combined to create basic graph patterns. SPARQL graph pattern matching is defined in terms of combining the results from matching basic graph patterns with RDF graphs. SPARQL enables users to query RDF graphs using required and optional graph patterns along with their conjunctions and disjunctions.

Ideally, using the SPARQL graph query language any user can query multiple RDF graphs simultaneously on the LOD cloud. This approach is often called ‘SPARQL query federation’ or ‘distributed SPARQL query processing’.^29,40,61,107^ While this approach is inspired from the relational database community, SPARQL query federation architectures leverage the advantages provided by the graphical, uniform syntax, and schema-less nature of RDF to achieve query federation with minimal effort. SPARQL query federation also differs from conventional ‘data warehousing’ approach, where data and knowledge is extracted from multiple sources, transformed to uniform schemas and entity notations, and loaded into a data warehouse. Moreover, SPARQL query federation architectures can be coupled with “intelligent” mechanisms (e.g., greedy algorithms, rule-based reasoning methods) for efficient source selection, query execution, and structured reasoning.^29,40,61,107,108^

Few benefits of such a Web-based SPARQL query federation approach over conventional approaches for data and knowledge integration (e.g., data warehousing) are enumerated below: [noitemsep]

1. Scalability: Easily deal with volume, variety and velocity of underlying sources.
2. Flexibility: Easily incorporate multiple remote sources during query processing and execution.
3. Exhaustivity: Easily retrieve all available and relevant knowledge related to a specific entity.
4. Mutability: No update mechanisms are required to handle modifications in remote sources.
5. Minimal technicality and redundancy: No requirements of downloading, transforming and storing content locally, no additional copies of the remote sources, minimal requirements of programming skills for most users, sharing of queries between projects that integrate similar sources.

## Opportunities and applications in biomedicine

Using examples from three main research domains: (1) pharmacology, (2) cancer biology, and (3) epidemics, we will provide our perspective on how Semantic Web technologies and the LSLOD cloud can tackle several challenges for big biomedical data and knowledge integration, endowed due to the: (i) the volume, velocity, variety, and veracity of biomedical data and knowledge, (ii) the heterogeneity across different sources, (iii) and the requirements of exhaustive biomedical entity reconciliation, and enable the discovery novel associations, often serendipitously, in the context of available knowledge.

### Drug discovery, drug repurposing, and drug safety

Currently, it costs US$2.87 billion (in 2013 dollars) for the discovery of a novel drug by a bio-pharma company, and there is bound to be exponential increase in these costs.^38^ Researchers are now looking for novel uses of drugs existing in the market, often called drug repurposing, to mitigate these costs.^39^ Federal regulators monitor the occurrence of adverse drug reactions (ADR) after the public release of a particular drug, often called pharmacovigilance or drug safety. ADRs are not always be detected during the clinical trials, and may also manifest due to drug–drug interactions in patients.^40^ ADRs are the 4th leading cause of death exceeded only by diabetes, AIDS, and pneumonia.^41^ The ever-rising cost of drug-related morbidity and mortality in the United States was estimated to be US$177.4 billion in 2000.^42^

For biomedical research pertaining to drug discovery, drug repurposing, and drug safety, biomedical researchers often need an aggregated summary on available data and knowledge for a specific Drug entity (e.g., Gleevec) or need to pose queries, an example of which was introduced earlier using Fig. 2. Moreover, drug-related data and knowledge feature collected from multiple sources can be pushed into automated informatics pipelines (e.g., protein–ligand molecular docking, matching drug and disease gene expression profiles, network-based systems pharmacology methods) for large-scale systematic analyses to determine potential drug repurposing candidates or drug–drug interactions.

Biomedical researchers have often used conventional methods to address the problem of integrating data and knowledge from multiple pharmacological sources. The Open PHACTS (Open Pharmacological Concept Triple Store) data warehouse exposes integrated content, harvested from several legacy databases and structured using a common vocabulary with normalized entity identifiers, through user-friendly software interfaces to accelerate drug discovery research.^43^ Himmelstein et al.^44^ manually integrated content from 29 different sources using a common data model to create a systems pharmacology network ‘HetioNet’ composed of different biological entities. Similarly, Li et al.^45^ generated a causal systems pharmacology network ‘CauseNet’ by manually integrating four sources: DrugBank,^5^ PharmGKB,^46^ KEGG,^22^ and the Comparative Toxicogenomics Database (CTD).^47^

A few of the foremost biomedical projects on the LSLOD cloud were related to publishing pharmacological data and knowledge on the Web (e.g., Linking Open Drug Data,^48^ Bio2RDF^27^). Whereas, there has been research in ‘downloading’ the pharmacological RDF graphs from multiple LSLOD sources and integrating the content ‘locally’, these research methods do not perform Web-scale semantic processing and integration. Noor et al.^49^ constructed a mechanism-based DDI knowledge warehouse by integrating LSLOD content at the pharmacokinetic, pharmacodynamic, and pathway interaction level, and used an inference engine to generate mechanistic explanations for DDIs. ReDrugS^50^ uses a data warehousing approach to integrate Bio2RDF Linked Data sources, and the integrated content is analyzed using a probabilistic graphical model to predict drug repurposing candidates for melanoma.

The above methods often entail redundancy of technical efforts (e.g., all approaches may integrate content ‘locally’ from DrugBank,^5^ PharmGKB^46^ and KEGG^22^), along with other disadvantages (Box 3). Most of the sources are already available on the LSLOD cloud for Web-scale data integration. Kamdar et al.^40^ generated a systems pharmacology network, similar to CauseNet, using a SPARQL query federation method PhLeGrA (Linked Graph Analytics in Pharmacology) over Bio2RDF sources, and used the network in signal detection algorithms to detect pharmacovigilance associations from the US FDA Adverse Event Reporting database (FAERS) with explanations on underlying biological mechanisms.

### Data and knowledge integration for cancer research

Biomedical researchers are interested to investigate the dysregulated biological mechanisms underlying the different cancer types, and to introspect and validate the diagnostic, prognostic, and therapeutic capabilities of different biomarkers in cancer patients on a personalized basis. For such research goals, it is often necessary to obtain the complete picture regarding the specific cancer typology. This often entails the use of systems biology approaches that integrate network biology knowledge (e.g., signaling, metabolic, regulator pathways), proteins and drugs data (e.g., structures, indications, side effects), ‘-omics’ data (e.g., gene expression, DNA methylation), and patients’ clinical data, environmental and nutritional data, etc.

Ding et al.^51^ emphasized the need for developing novel approaches that investigate somatic mutations (i.e., genetic alterations propagated through cell division but are not inherited by children) in cancer genomes collectively, in conjunction with knowledge in gene sets (e.g., Gene Ontology^7^ annotations) or biological pathways and interaction networks (e.g., KEGG,^22^ Reactome,^52^ protein–protein interaction data from BioGrid,^53^ STRING,^54^ iRefIndex,^55^ protein–DNA interaction data from ENCODE^56^). Most of these sources are already available on the LSLOD cloud for querying. Apart from outperforming single-gene tests (i.e., just determining whether a mutation in the gene is significantly greater in cancer patients), such systems biology approaches can enhance our understanding of somatic mutations, decipher disease mechanisms and also aid in repurposing existing drugs for treatment toward different cancer types (e.g., as proposed by Turanli et al.^57^ for prostrate cancer). Moreover, knowledge that is readily available along with the genomics and clinical data of a patient, can aid the physician to make better clinical decisions (e.g., whether or not to prescribe a particular drug, such as Temozolomide, given a genomic alteration, such as a CpG methylation, in the cancer patient).

Semantic Web technologies and LSLOD resources are ideal for enabling Web-scale data integration for cancer research. Biomedical researchers have indeed utilized the OWL knowledge representation language to develop several cancer-related ontologies (e.g., National Cancer Institute Thesaurus (NCIT),^8^ a popular reference terminology, to represent cancer data across different research centers, Common Terminology Criteria for Adverse Events^58^ to capture adverse events observed in cancer therapy clinical trials, NanoParticle Ontology^59^ to characterize nano-materials used in cancer diagnosis and therapy, Radiation Oncology Ontology^60^ to map radiation data across clinical databases).

**Fig. 3:**
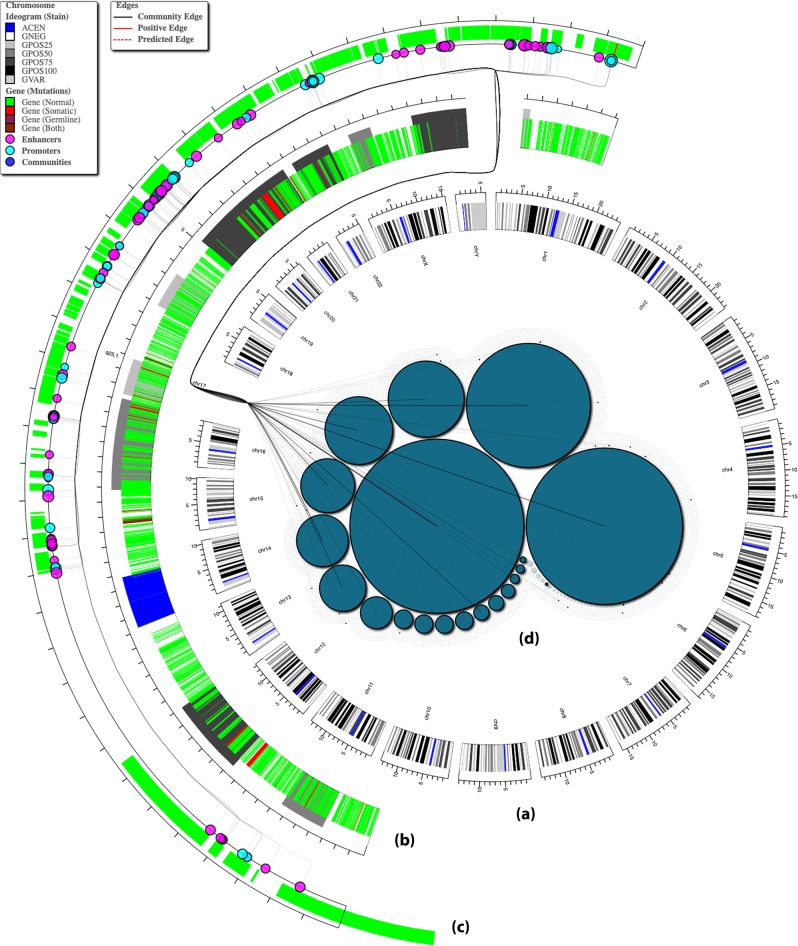
Interacting with Big Linked Cancer Data through the GenomeSnip visualization perspective. The Linked TCGA project provides several different visualization perspectives for biomedical researchers to explore and visualize integrated content from the following LSLOD data sources: (i) MESH, (ii) HGNC, (iii) KEGG, (iv) PubMed, (v) UniProt, and (vi) Linked TCGA. The GenomeSnip perspective provides an aggregative circular visualization of the human genome, and allows the user to interactively explore different genomic regions at different scales—**a** chromosome, **b** ideogram, and **c** gene and other regulatory regions (e.g., enhancers). **d** Relations (protein–protein interactions, gene co-mentions, etc.), as well as communities of genes or genomic regulatory regions, as detected by a community-detection or clustering algorithm, can also be visualized. The GenomeSnip perspective is available online at http://onto-apps.stanford.edu/genomesnip

Kamdar et al.^61^ developed a visual query system ReVeaLD (Real-time Visual Explorer and Aggregator of Linked Data) that used a query federation architecture for querying 80+ LSLOD sources relevant to cancer research. ReVeaLD enabled cancer researchers to formulate SPARQL queries (e.g., Fig. 2) visually, and to then filter and transfer the retrieved data (e.g., a set of retrieved molecular structures) for further analysis in ‘in silico’ protein–ligand docking experiments.^62^ ReVeaLD was also used to integrate publicly-available knowledge on proteins and existing protein–protein interactions from multiple sources, such as BioGrid,^53^ CORUM,^63^ pFam,^64^ and the Human Protein Atlas,^65^ and using the knowledge features in machine learning algorithms to discover novel protein–protein interactions.^66^ Saleem et al.^29^ published the genomics and clinical datasets of cancer patients in The Cancer Genome Atlas project as Linked Data (Linked TCGA) and showcased the use of a query federation architecture to query Linked TCGA in conjunction with other sources on the LSLOD cloud. The Linked TCGA project and the associated multi-faceted visualization perspectives are described in more detail in Box 4 (Fig. 3).

### Infectious diseases and epidemics

While cancer research and pharmacological research typically deal with data and knowledge of great volume and variety, research pertaining to infectious diseases and epidemics is often characterized by data and knowledge of great velocity. Web-scale semantic processing can benefit several aspects of informatics approaches that analyze social media streams for monitoring epidemics (e.g., annotating dynamically generated content from diverse geographical regions with concepts from the LSLOD sources, and using the annotated content for reasoning and inference). Apart from social media streams, there is relevant data and knowledge from other research sources pertaining to infectious diseases: sequencing of microbial genomes and proteomes, experimental assays to identify ligands that target select proteins for therapy, publications that document these experiments, etc.

**Fig. 4:**
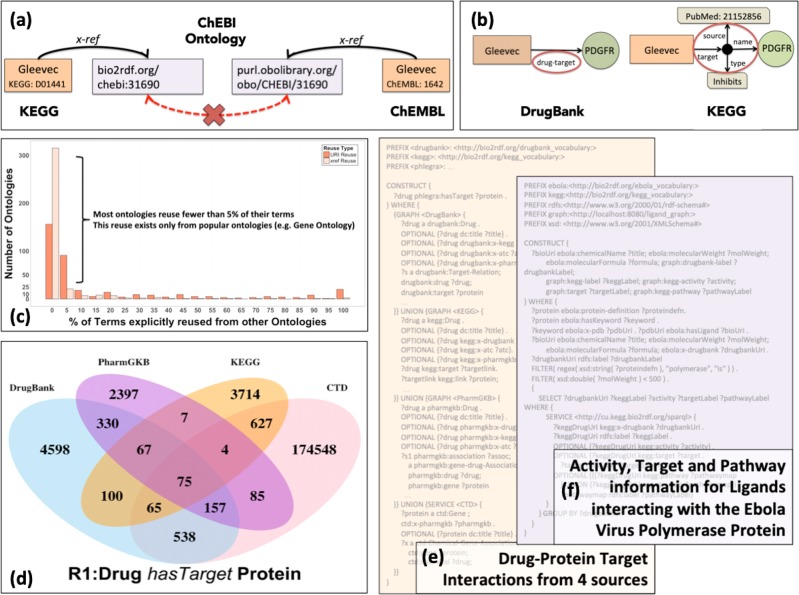
Challenges in consuming LSLOD content for biomedical applications. **a** Different LSLOD sources may use different URI representations for the same entity (e.g., different ChEBI URIs http://bio2rdf.org/chebi:31690 and http://purl.obolibrary.org/obo/CHEBI/31690 for the entity Gleevec). Hence, link traversal or query federation methods are not able to integrate content from KEGG and ChEMBL RDF graphs even when they have ‘x-ref’ links to the ‘similar’ ChEBI entity. **b** Different RDF graphs may use different semantics (e.g., drug-target and target). Different graph patterns may be used to depict the same relation, while capturing additional details. **c** Through a systematic analysis of biomedical ontologies in BioPortal repository, we determined that while a significant overlap of content exists across biomedical ontologies, most ontologies reuse less than 5% of their terms with several ontologies using incorrect term URIs (Graph generated from data presented in Kamdar et al.^81^). **d** Unique drug–protein target interactions may exist across different data and knowledge sources, since these sources are published with different methods and intentions (Figure used with permission under a CC-BY Attribution 4.0 International License from Kamdar et al.^40^). **e** Real-world SPARQL query to retrieve drug–protein target interactions from four different LSLOD sources–DrugBank, KEGG, PharmGKb and Comparative Toxicogenomics Database. **f** Real-world SPARQL query to retrieve activity, target, and pathway information for ligands interacting with the Ebola virus polymerase protein

Nolin et al.^67^ generated a mashup of time-course microarray gene expression results with protein–protein interaction data from Bio2RDF sources to understand the infection of human macrophages with human immunodeficiency virus 1 (HIV-1). The 2013–2016 Ebola Virus epidemic had a cumulative death rate of 41% and 24,000 reported cases (as of 20 March 2015). Kamdar et al.^68^ developed a linked mashup Ebola-KB that integrates publicly-available knowledge, pertaining to the Ebola Virus Disease, from several Linked Data sources such as Gene Ontology,^7^ MEDLINE,^12^ Protein Data Bank,^69^ etc. Questions, such as “Retrieve knowledge from KEGG and DrugBank on small molecule ligands which bind to EBOV protein Polymerase”, can be answered by executing SPARQL queries against the Ebola-KB linked mashup in conjunction with the LSLOD cloud (see Fig. 4f).

Semantic Web technologies and LSLOD sources can also aid biomedical researchers to gain insights into drug-related epidemics in the United States. To understand the genetic basis of nicotine dependence, Sahoo et al.^70^ developed a semantic mashup by integrating genetic resources Entrez Gene^71^ and Homologene,^72^ with pathway resources KEGG,^22^ Reactome,^52^ and BioCyc,^73^ structured using the BioPAX ontology.^74^ Approximately 50,000 individuals have died due to opioid-related overdoses in 2017 alone—this count has tripled in the last decade. Hypothetically, a researcher can analyze clinical data and social media data can be analyzed in conjunction with LOD resources, such as drug ontologies (e.g., ATC^75^ and RxNorm), demographic and geographic databases (e.g., Wikidata^76^ and DBPedia^77^), patient symptoms terminologies (e.g., MEDDRA), disease ontologies (e.g., ICD-10), to determine opioid prescription and usage patterns and predict whether a patient or a user demonstrates opioid abuse behavior.

Box 4 Web-scale Linked Cancer Data Cancer systems biology approaches often rely on ‘-omics’ and clinical datasets of cancer patients, few of which are also available publicly. For example, the Cancer Genome Atlas (TCGA) publishes the genomics (e.g., DNA methylation, exon expression, miRNA expression) and clinical data of individuals, categorized under different cancer types.

Semantic Web developers have published publicly-available cancer genomics datasets as Linked Data to enable the development of analytical pipelines for automated analyses. Developing such analytical pipelines over genomics data often involves redundant, non-trivial, and difficult tasks for most biomedical researchers, such as downloading and preprocessing large data archives, feature extraction and linkage to existing biological knowledge. Under the Linked Cancer Genome Atlas (Linked TCGA) project,^29^ raw TCGA data for 27 different cancer types is preprocessed, converted, and published as Linked Data in order to facilitate the querying and live integration of these cancer datasets via remote SPARQL query processing. Linked TCGA data is also linked with content from several existing LSLOD sources that contain relevant knowledge on biological pathways (e.g., KEGG^22^), proteins (e.g., UniProt^6^), and diseases (e.g., Diseasome). Biomedical publication abstracts from the PubMed MEDLINE^13^ repository are processed through a natural language processing pipeline and named entities (i.e., proteins, cancer types, and drugs) are annotated using concepts from the LSLOD cloud. Hence, unstructured publications are made available for querying along with structured cancer ‘-omics’ and clinical data, as well as knowledge from public knowledge bases.

The Linked TCGA project also provides several different visualization perspectives so that biomedical users can visualize and explore integrated content from Linked TCGA and several other sources on the LSLOD cloud without formulating extensive federated SPARQL queries. For example, the GenomeSnip^104^ perspective (shown in Fig. 3) allows the user to interact with an aggregative circular visualization of the human genome and explore genomic regions (e.g., ideogram, gene, regulatory region, or individual single nucleotide polymorphism—SNP) and relationships (e.g., those genes that co-occur in the same publication or that transcribe proteins involved in the same pathway or disease). Communities of genes identified using a community-detection or a clustering algorithm can also be visualized. Saleem et al.^29^ showcase how biomedical users can retrieve and visualize cancer-related publications associated with a particular MESH topic (e.g., Clone Cell) or a gene (e.g., GRBB2) using a Network Explorer visualization perspective, which features a highly dense, force-directed network linking the different tumor typologies, genes, publications, and MESH topics. TCGA genomic datasets (i.e., DNA methylation and exon expression) of the cancer patients can be visualized against the human genome, and the GenomeSnip and the Network Explorer perspectives can be used to further filter and explore the data interactively on the Web (e.g., visualize the TCGA genomic data for a particular Gene entity or a set of Gene entities mentioned in a given Publication, or Gene entities that are present in the same cluster).

The Linked TCGA project demonstrates the true utility of Semantic Web technologies and Life Sciences Linked Open Data for Web-scale semantic processing and data integration. Content (structured and unstructured) from several data and knowledge sources is integrated and made available for the biomedical researcher to interactively explore, as well as to use the integrated content for analysis in machine learning methods.

## Challenges in using LSLOD for biomedical applications

Significant resources have been invested in publishing biomedical data and knowledge on the Web to create the LSLOD cloud. However, to the best of our knowledge, outside of a few research groups (including ours), there are not many biomedical applications that demonstrate Web-scale Semantic Processing and Data Integration by consuming LSLOD in a decentralized fashion (i.e., querying directly on the Web). As seen through the examples in the previous section, there are significant benefits of using query federation and graph-based methods over conventional methods for tackling the integration bottlenecks in different research areas of biomedicine. However, decentralized biomedical applications that actually exist (e.g., Linked TCGA, ReVeaLD) have generally seen minimal adoption by the broader biomedical research community.

The biomedical community has indeed been using the Web Ontology Language (OWL) for the development of large biomedical vocabularies and ontologies for a long time.^11^ These resources comprise a major portion of the LSLOD cloud. However, biomedical ontologies are typically used in “closed” systems or centralized applications (i.e., data warehouses), and they are not queried over the Web in most cases. For example, the Gene Ontology (GO),^7^ arguably the biomedical ontology with the highest impact in the community, is widely used for Gene Set Enrichment Analysis (GSEA) applications. However, these applications often rely on locally-downloaded versions of Gene Ontology.

Publishers often mention the “potential” of Linked Data to solve data integration challenges, but only showcase use cases where the LSLOD sources are queried in a controlled environment.^29,68^ However, most biomedical researchers do not retrieve fruitful results (or any useful results in most cases) when they query against the LSLOD sources in the wild. We have identified few of the most important challenges faced while using content directly from the LSLOD cloud in biomedical applications.

### Accessibility and availability

The accessibility and availability of LSLOD sources are two of the major reasons why data and knowledge within the LSLOD cloud cannot be queried and consumed by biomedical researchers. According to the statistics and metadata descriptions at lod-cloud.net, the 2017 version of the Linked Open Data (LOD) cloud had 1281 sources, out of which only 50% (646 sources) had a functional Linked Data access point (i.e., a RDF data dump or a SPARQL endpoint).^78^ In addition, it is relatively easy that online complex queries (e.g., queries with multiple non-selective joins) may incur in timeouts, given the limited allocated resources of public SPARQL endpoints.

If the LSLOD sources do not have high availability then the research and development of Semantic Web query federation methods and tools in biomedicine is severely impacted.

The liveliness and ‘freshness’ of LSLOD sources depends heavily on the continued support and interest of their maintainers (who are often from academia). Once the maintainers leave the project, often, the SPARQL endpoints are not updated anymore and may stop being available. Sustainable access on the Web with regular updates, in compliance with the Linked Data principles, has simply not been a priority for various data and knowledge providers. For example, the highly-available SPARQL endpoint of BioPortal (http://sparql.bioontology.org/) is not frequently updated. Similarly, updated Bio2RDF RDF dumps, available only via a Javascript-based page (http://download.bio2rdf.org/#/), cannot be easily crawled by machines. Academia-based Linked Data resources (e.g., DERI Health Care and Life Sciences workbench from National University of Ireland Galway–http://bit.ly/2XmZQUX), which are maintained by only one research group and are often reliant on the funding sources, cease to exist once the funding periods end. Several valuable LSLOD sources (e.g., RDF graphs from Linking Open Drug Data projects) are no longer available.

### Semantic heterogeneity

Semantic heterogeneity is a natural consequence of the independent creation and evolution of autonomous data sources and ontologies that are tailored to the requirements of the domain and application system they serve.^79^ The semantic heterogeneity across the different biomedical ontologies and Linked Data resources is another major reason for the lack of usage of LSLOD content in biomedical applications directly from the Web. The fourth Linked Data principle (Box 1) emphasizes the correct reuse of existing vocabularies and ontologies, as well as linking to entities that already exist on the LSLOD cloud using the exact Uniform Resource Identifiers (URIs). Automated traversal and data integration across LSLOD sources only work if the sources are linked using exactly correct URIs for the same terms consistently (i.e., as an analogy, navigating on the Web only works when the http://hyperlinks are correctly specified). In practice, this trivial requirement is often not satisfied.

#### Intent for reuse

Publishers reuse inconsistently (and often, incorrectly) URIs used to represent different biomedical entities. For example, Kamdar^80^ found that different LSLOD sources refer to the same UniProt^6^ Protein entity (e.g., Q9UJX6) using the following different UniProt URI representations:

(i) http://purl.obolibrary.org/obo/UniProt:, (ii) http://bio2rdf.org/uniprot:, (iii) http://purl.uniprot.org/uniprot/, and (iv) http://identifiers.org/uniprot/.

This issue creates a significant burden for biomedical application developers, who use the LSLOD cloud for Web-scale data integration but will be unaware of all these URI representations across different datasets. For example, as shown in Fig. 4a, a biomedical researcher wishes to retrieve and integrate content from KEGG and ChEMBL RDF graphs^35^ (a hypothetical query can be “Retrieve biochemical activities of compounds that target proteins in Apoptopic Signaling pathway”). However, while the compounds in both these RDF graphs are mapped to terms in the ChEBI ontology,^82^ the URIs are different. Hence, the researcher cannot navigate or query the two RDF graphs in an integrated fashion (manually, or using conventional query federation methods). This issue, called “intent for reuse” (i.e., publishers wish to refer to the same biomedical entity, but use slightly different URI representations), is manifested across many biomedical ontologies, as documented by Kamdar et al.^80,81^

#### Lack of reuse

In Fig. 4a, the different compound entities are still mapped to similar terms (albeit different URIs) from a common ontology (e.g., ChEBI ontology^82^). In many cases, instead of using common vocabularies or ontologies (e.g., from BioPortal repository) to represent the classes and properties in their RDF graph schemas, data publishers use their own custom vocabularies to generate RDF data.

As shown in Fig. 4b, different URIs may be used to represent the relations of type Drug $\mathop{\longrightarrow}\limits_{{has - target}}$ Protein in different sources (e.g., drugbank:drug-target and kegg:target). Moreover, completely different graph patterns may be used to capture these relations. For example, in Fig. 4b, the object of the kegg:target triple is a ‘blank node’. A blank node is a specialized RDF resource that facilitates the representation of complex relations and attributes with higher level of granularity (e.g., type of interaction between Gleevec and PDGFR), provenance information (e.g., publication that documents the interaction between those entities), or even lists of resources.^17^ Dealing with such blank nodes during Web-scale query federation and integration is inherently difficult. To explain simplistically, in Fig. 4b, the protein target of drug Gleevec is located one hop away while navigating DrugBank RDF graph, whereas the protein target is located two hops away while navigating the KEGG RDF graph.

“Actual and Correct Reuse” as advocated by the Linked Data principles and by various ontology engineering methodologies^83^ is generally much less across biomedical ontologies and Linked Data sources in the LSLOD cloud. Kamdar et al.^81^ found that while significant term overlap exists across biomedical ontologies in the BioPortal repository, most ontologies reuse less than 5% of their terms and ontology developers just use completely different representations (or show an “intent for reuse”, as presented in the previous section). This result is shown in Fig. 4c. This lack of reuse of concepts and properties from existing vocabularies and ontologies is also observed across most biomedical Linked Data sources.^80^

### Learnability and usability of Semantic Web technologies

There is a steep learning curve to understand and use Linked Data and Semantic Web technologies for biomedical researchers, who will use the content for scientific research and discovery. The architectural and structural issues with the LSLOD cloud, discussed in the preceding sections, make it more difficult for biomedical researchers to use the LSLOD cloud for Web-scale data integration.

The assembly of federated SPARQL queries to retrieve information necessary for bioinformatics analysis poses a high cognitive entry barrier, is time-consuming and a highly technical process. The direct consequence of semantic heterogeneity on Web-scale semantic processing and integration is that biomedical users need to formulate exhaustive SPARQL queries using conventional query federation methods, and to be aware of the different URI representations and data representation schemas used in the LSLOD cloud. For example, a biomedical researcher may wish to retrieve drug–protein target relations from multiple sources (e.g., DrugBank, KEGG, PharmGKB, and CTD), since unique relations may exist in each source (e.g., drug–protein target relations may be curated from drug product labels or from literature) as shown in Fig. 4d (taken from Kamdar et al.^40^). However, he/she has to formulate a complex SPARQL query with >20 triple patterns for this task (Fig. 4e). It is ‘almost’ impossible to generate a systems pharmacology network (composed of multiple relation types) as presented by Himmelstein et al.^44^ or Li et al.^45^ using most ‘conventional’ query federation methods over the current LSLOD cloud. Figure 4f shows another example of a complex (yet relevant) federated query across the Ebola-KB linked mashup and the KEGG LSLOD source.^68^

It is probably naive to expect that, for their data and knowledge integration needs, most biomedical researchers will formulate sophisticated SPARQL queries over heterogeneous LSLOD sources that have limited availability, without minimal automated support. There is a dire need for HCI-inspired applications and visualizations over the LSLOD cloud to make it easy for biomedical researchers to query and explore LSLOD content (e.g., Linked TCGA visualizations discussed in Box 4), as well as to make it easy for data and knowledge publishers to discover and reuse existing LSLOD content in a correct way, hence reducing the spread of semantic heterogeneity.^81,84^

## The silver lining of the LSLOD cloud

The major issues, presented in the previous section, which hinder the use of Linked Open Data for Web-scale semantic processing and data integration in biomedicine may present a bleak picture on the future of the LSLOD cloud and Semantic Web technologies in general.

For most biomedical projects that use Semantic Web technologies for reasoning and inference, the most common solution to the semantic heterogeneity problem is to use a data warehousing approach (e.g., OpenPhacts,^43^ ReDrugs^50^), where all data is transformed under a common schema and using a uniform set of entity notations. There are other significant advantages of data warehousing over query federation even in a Linked Open Data scenario—data cleaning, privacy, trust, data preservation, and to a certain extent, indexing and querying.^85^ Data warehouses indeed require a lot of centralization and maintenance, and need to be updated when the underlying content changes. Data warehousing approaches require significant resources and can only be implemented as part of a consortium or by companies. However, the issues of network latency, the availability and accessibility of SPARQL endpoints, as well as the quality of remote data sources, can easily be remedied through a data warehousing approach.

In this section, we assert that there is definitely a silver lining to the LSLOD cloud and the Semantic Web community is actively working on technical solutions to address each of these issues. In this section, we briefly touch on a few examples of such technical solutions.

### Accessibility and availability

As a part of a solution path to one of the main handicaps for further adoption, monitoring frameworks, such as SPARQLES^86^ or the Dynamic Linked Data Observatory,^87^ are essential to assess the parts of the LSLOD cloud that are still “alive”. Preservation efforts, such as the LOD Laundromat project,^88^ are also good starting points to crawl and provide archives of existing datasets. There has been recent development on scalable off-the-shelf tools that can alleviate some of burden of the Linked Data publisher. In particular, a combination of (i) RDF graphs uploaded as HDT^89^ (Header-Description-Triples), a highly compressed and queryable RDF format, as well as (ii) Triple Pattern Fragments endpoints^90^ as the standard access method for LSLOD sources, significantly reduces both infrastructural and maintenance needs. Improving the availability of public SPARQL endpoints is also an area of active research (e.g., research on alternative query strategies for federated queries^91^ and better load balancing between client and server.^92^)

There are two main LSLOD sources that are exemptions related to the Web-based availability and “freshness” of biomedical semantic resources: the Gene Ontology (GO)^7^ and the Unified Medical Language System (UMLS).^24^ In our opinion, the success of the Gene Ontology is, in part, due to the following main factors: (1) A dedicated and a very active development team with continuous funding has maintained it over several years; (2) A strong community of domain users from different areas has been actively built around it, and their requirements serve as the main impetus the development process; (3) The ontology itself has an exemplary documentation on its usage in applications targeted to domain users, and on the processes for building and maintaining it; (4) A principled approach was used for developing the ontology; (5) Automated pipelines are used to check and ensure the quality of the ontology. This is also true for UMLS-based semantic resources (e.g., SNOMED CT terminology^93^). Public SPARQL endpoints of DBPedia^77^ and Wikidata^76^ have started registering ≈99% uptime, as monitored using the SPARQLES framework. The providers of other LSLOD resources can definitely learn from these projects.

### Semantic heterogeneity

Hybrid approaches (i.e., approaches that combine query federation with initial processing and transformation) have been successful in the pharmacological research community for heterogeneous data integration. OHDSI collaborative^94^ (Observational Health Data Science Initiative) for observational drug safety, DisQover^95^—a commercial platform for semantic search in life sciences, and even the Open PHACTS data warehouse,^43^ extract and transform content uniformly using a common data model and entity notations, and publish the transformed content as Linked Data interfaces. If such approaches can be combined with methods to detect changes and evolution in LSLOD resources (e.g., COnto–Diff^96^), then we can have sustainable Web-scale data integration solutions.

Novel frameworks, such as Debattista et al.^97^ and Kamdar,^80^ can automatically provide fine-grained quality metrics to mitigate availability and semantic heterogeneity problems. In addition to these initiatives, the LSLOD cloud itself needs to be a “live” environment and providers who do not provide minimal availability (i.e., less than 99% uptime) or desired quality should be notified. Conversely, LSLOD consumers should be notified of important changes in a dataset. Decentralized protocols using the same technologies, such as Linked Data Notifications,^98^ can serve as communication vehicles for such synchronization.

### Usability and learnability

Emergent industry-strength solutions for Linked Data-related tasks, such as extraction and mapping (e.g., Rules Markup Language—R2ML for rules interchange between different systems^99^), validation (e.g., Shapes Constraint Language—SHACL for validating the structure of RDF graphs^100^), integration of legacy systems (e.g., Ontology Based Data Access—OBDA for using an ontology to access legacy relational databases^101^), and consumption (e.g., efficient RDF triplestores^102^), can assist towards addressing usability issues and maintaining documentation standards.

Mappings-based query federation methods^40,61,62,80^ can slightly alleviate the challenge of semantic heterogeneity. Biomedical researchers can formulate SPARQL queries using elements from a domain-specific common data model. These SPARQL queries are then transformed “under-the-hood” to source-specific SPARQL queries, through mappings between the elements from the domain-specific data model and the elements of from the data representation schemas of the remote LSLOD sources.

In the example shown below, the triple pattern composed using some domain-specific common data model is transformed to two sets of triple patterns for two different LSLOD sources–DrugBank and KEGG. This transformation is guided through mappings between the elements of the domain-specific common data model and the elements observed in the schemas of DrugBank and KEGG.

$${\mathbf{Drug}}\mathop{\longrightarrow}\limits^{{hasTarget}}{\mathbf{Protein}} = \left\{ {\begin{array}{*{20}{l}} {{\mathbf{Drug}}\mathop{\longleftarrow}\limits^{{drug}}{\mathrm{Target}} - {\mathrm{Relation}}\mathop{\longrightarrow}\limits^{{target}}{\mathbf{Protein}}} \hfill & {{\kern 1pt} {\mathrm{if}}\,(DrugBank)} \hfill \\ {{\mathbf{Drug}}\mathop{\longrightarrow}\limits^{{target}}{\mathrm{:}}_ - {\mathrm{blank}}\mathop{\longrightarrow}\limits^{{link}}{\mathbf{Protein}}} \hfill & {{\kern 1pt} {\mathrm{if}}\,(KEGG)} \hfill \end{array}} \right.$$

Using such mapping rules and a mappings-based query federation method, the SPARQL query shown in Fig. 4e can be formulated with ≈5 triple patterns and the biomedical researcher can retrieve the drug–protein target relations from four sources.^80^ Hence, the researcher does not need to be familiar with the various representation schemas used in the LSLOD cloud to formulate SPARQL queries. However, the mappings need to be validated, often manually, by Semantic Web experts. If the mapping rules can be validated, either autonomously (e.g., using Shape Expressions^103^) or using a visualization interface by a domain expert, then such methods can also be sustainable for Web-scale data integration using Linked Open Data without the shackles of centralization using data warehousing methods.

Finally, more applications that enable biomedical researchers to formulate SPARQL queries using visual interactions (e.g., ReVeaLD^61^), applications that generate multi-faceted visualizations (e.g., Linked TCGA dashboard^29,104^ and Ebola-KB dashboard^68^) for biomedical researchers to explore the integrated data, abstracting SPARQL and RDF entirely, or more studies that analyze user interactions with LSLOD sources (e.g., Kamdar et al.^61,84^) are definitely required for increasing the adoption of LSLOD and Semantic Web technologies in the biomedical research community.

Most of the aforementioned issues can be further ameliorated by following standard best practices, such as the recent FAIR data principles^105^ (findable, accessible, interoperable, reusable) and the ‘Data on the Web Best Practices’,^106^ both fostering the creation of a self-sustainable ecosystem. Additionally, common-sense good practices suggest to provide: (1) better metadata descriptions of the datasets; (2) better documentation and provision of sample queries for usage of the datasets; (3) better support for enabling reuse of existing vocabularies; and (4) better support for the use of developer-friendly formats (e.g., JSON), with a toolchain maintained by an active and a broader community.

Finally, while redundancy of technical efforts is not ideal, several research groups may often wish to keep their data, searches, and inferences private. While biomedical data and knowledge sources form the largest portion on the Linked Open Data cloud, a lot of biomedical data is, and will always remain, in closed systems (e.g., electronic health records). Hence, we envision that centralized and decentralized approaches will always have to co-exist and complement each other in the biomedical ecosystem to tackle complex problems. Ideally, researchers can avail the benefits of Linked Open Data for Web-scale integration of public data and knowledge, slices of which can then guide advanced searches and inferences over the private data and knowledge stored in their centralized data warehouse.

## Conclusion

The biomedical data landscape is fragmented with several isolated data and knowledge sources existing on the Web. These biomedical sources may use varying formats, schemas, syntaxes, entity notations, and modes of access, which increase the logistical and technical challenges related to data and knowledge integration for most biomedical researchers. While there is hope that the next generation of artificial intelligence methods can augment human intelligence for achieving better clinical outcomes for patients on a personalized level, for increasing our understanding of living organisms, and for enhancing the quality of biomedical research, we lack scalable, intelligent infrastructures that can generate integrated content for use in these methods. This eventually leads to minimal scalability, minimal flexibility, minimal reproducibility, and increased redundancy of data integration efforts across different research groups that may simultaneously be working on similar biomedical problems.

In this paper, we have put forth our perspective on how Semantic Web technologies and the Life Sciences Linked Open Data (LSLOD) can enable the development of such scalable intelligent infrastructures for Web-scale semantic processing and data integration in biomedicine. We have showcased a real-world example pertaining to querying, retrieval, and integration, of data and knowledge from diverse biomedical sources. We have also discussed the main challenges: (i) accessibility and availability, (ii) semantic heterogeneity, and (iii) usability and learnability, which hinder the use and consumption of content from the LSLOD cloud. We present a few technical solutions from the Semantic Web community that hope to convince biomedical researchers, that while these challenges provide a bleak outlook on the future of the LSLOD cloud, there is indeed light at the end of the tunnel. In an ideal state of the LSLOD cloud, the opportunities for data and knowledge integration in pharmacology, cancer research, infectious diseases, and several other biomedical domains, will eventually be realized in biomedicine, leading to better clinical outcomes and enhancing the quality of biomedical research.

## Data Availability

Several data and knowledge sources in the Life Sciences Linked Open Data cloud were systematically evaluated through different studies and the findings are summarized in this perspective paper. These LSLOD sources (along with links to available SPARQL endpoints or RDF data dumps) are listed at the GitHub repository https://github.com/maulikkamdar/LSLODQuery.

## Code availability

All scripts used to analyze the LSLOD cloud are made available under the MIT License in the GitHub repository https://github.com/maulikkamdar/LSLODQuery. The biomedical applications developed by our group and discussed in this paper are available under the Creative Commons CC BY-NC-SA License at https://github.com/maulikkamdar/OntoApps-Stanford.
